# Optimization and Improvement of qPCR Detection Sensitivity of SARS-CoV-2 in Saliva

**DOI:** 10.1128/spectrum.04640-22

**Published:** 2023-04-25

**Authors:** Hui-Ying Ko, Yao-Tsun Li, Ya-Yuan Li, Ming-Tsai Chiang, Yi-Ling Lee, Wen-Chun Liu, Chun-Che Liao, Chih-Shin Chang, Yi-Ling Lin

**Affiliations:** a Institute of Biomedical Sciences, Academia Sinica, Taipei, Taiwan; b Biomedical Translation Research Center, Academia Sinica, Taipei, Taiwan; Emory University School of Medicine

**Keywords:** SARS-CoV-2, COVID-19, saliva, RT-qPCR, diagnosis

## Abstract

Coronavirus disease 2019 (COVID-19), caused by severe acute respiratory syndrome coronavirus 2 (SARS-CoV-2), has been a major public health threat globally, especially during the beginning of the pandemic in 2020. Reverse transcription-quantitative PCR (RT-qPCR) is utilized for viral RNA detection as part of control measures to limit the spread of COVID-19. Collecting nasopharyngeal swabs for RT-qPCR is a routine diagnostic method for COVID-19 in clinical settings, but its large-scale implementation is hindered by a shortage of trained health professionals. Despite concerns over its sensitivity, saliva has been suggested as a practical alternative sampling approach to the nasopharyngeal swab for viral RNA detection. In this study, we spiked saliva from healthy donors with inactivated SARS-CoV-2 from an international standard to evaluate the effect of saliva on viral RNA detection. On average, the saliva increased the cycle threshold (*C_T_*) values of the SARS-CoV-2 RNA samples by 2.64 compared to the viral RNA in viral transport medium. Despite substantial variation among different donors in the effect of saliva on RNA quantification, the outcome of the RT-qPCR diagnosis was largely unaffected for viral RNA samples with *C_T_* values of <35 (1.55 log_10_ IU/mL). The saliva-treated viral RNA remained stable for up to 6 h at room temperature and 24 h at 4°C. Further supplementing protease and RNase inhibitors improved the detection of viral RNA in the saliva samples. Our data provide practical information on the storage conditions of saliva samples and suggest optimized sampling procedures for SARS-CoV-2 diagnosis.

**IMPORTANCE** The primary method for detection of SARS-CoV-2 is using nasopharyngeal swabs, but a shortage of trained health professionals has hindered its large-scale implementation. Saliva-based nucleic acid detection is a widely adopted alternative, due to its convenience and minimally invasive nature, but the detection limit and direct impact of saliva on viral RNA remain poorly understood. To address this gap in knowledge, we used a WHO international standard to evaluate the effect of saliva on SARS-CoV-2 RNA detection. We describe the detection profile of saliva-treated SARS-CoV-2 samples under different storage temperatures and incubation periods. We also found that adding protease and RNase inhibitors could improve viral RNA detection in saliva. Our research provides practical recommendations for the optimal storage conditions and sampling procedures for saliva-based testing, which can improve the efficiency of COVID-19 testing and enhance public health responses to the pandemic.

## INTRODUCTION

The coronavirus disease 2019 (COVID-19) pandemic has presented a significant public health challenge in the current century, placing an extensive burden on health care systems globally (1, 2). Severe acute respiratory syndrome coronavirus 2 (SARS-CoV-2), the virus responsible for COVID-19, is evolving and generating variants with increased transmissibility and immune evasion mutations (3, 4). COVID-19 vaccination has played a major role in protecting against severe symptoms and death, but frequent breakthrough infections and reinfections warrant collateral control measures, for example, isolation and quarantine, to stop the transmission (5, 6).

Effective COVID-19 control measures depend on tests that are accessible and have a short turnaround time. Real-time quantitative PCR (qPCR) has been extensively employed as a diagnostic and screening modality for COVID-19 since its inception in 2019. Different types of SARS-CoV-2 samples have been collected for qPCR, including samples collected from nasopharyngeal swabs, nasal swabs, oropharyngeal swabs, throat swabs, and saliva (S) (7–9). As for other upper respiratory viral diseases, the nasopharyngeal swab is currently considered a standard sample source for a COVID-19 diagnosis (10, 11).

Although sampling by nasopharyngeal swab could increase the sensitivity of detection (12–15), problems arise in conducting routine or mass testing. First, standard nasopharyngeal swab sampling requires medical professionals. Insufficient qualified medical staff will decrease productivity and thereby increase the turnaround time. Second, the practice can easily ignite sneezing, which generates aerosols, risking infection of people in close proximity. Finally, the discomfort rendered by the swab mentally discourages people from voluntarily taking the test. These issues could hamper existing control strategies if only nasopharyngeal sampling is allowed for qPCR.

Saliva sampling has been a convenient alternative to nasopharyngeal swab testing for COVID-19 because of the presence of viral RNA in patient saliva (12, 13, 16). Despite concerns over the sensitivity of saliva testing, meta-analyses comparing the sensitivity of qPCR using nasopharyngeal swabs and saliva samples indicated that the sensitivity of saliva-based qPCR is comparable (15, 17) or slightly lower (7, 14) than that of nasopharyngeal-based qPCR. Despite the apparent advantages and feasibility of collecting saliva for SARS-CoV-2 detection, the direct effect of saliva on viral RNA remains unclear. Understanding the effect of saliva on viral RNA and the factors involved is critical to further optimizing the diagnostic procedure.

In this study, to investigate the effect of saliva on viral RNA, we collected saliva samples from healthy individuals and added inactivated SARS-CoV-2 viruses (WHO international standard 20/146) to the saliva. The saliva-treated SARS-CoV-2 RNA levels were closely examined at different storage temperatures and incubation periods. With the aims of improving the sample collection procedure and searching for ways to increase viral RNA integrity, our results provide clues for a more sensitive and standardized saliva-based qPCR method for SARS-CoV-2 detection.

## RESULTS

### Reduced SARS-CoV-2 RNA detection in saliva samples compared to the VTM control.

**FIG 1:**
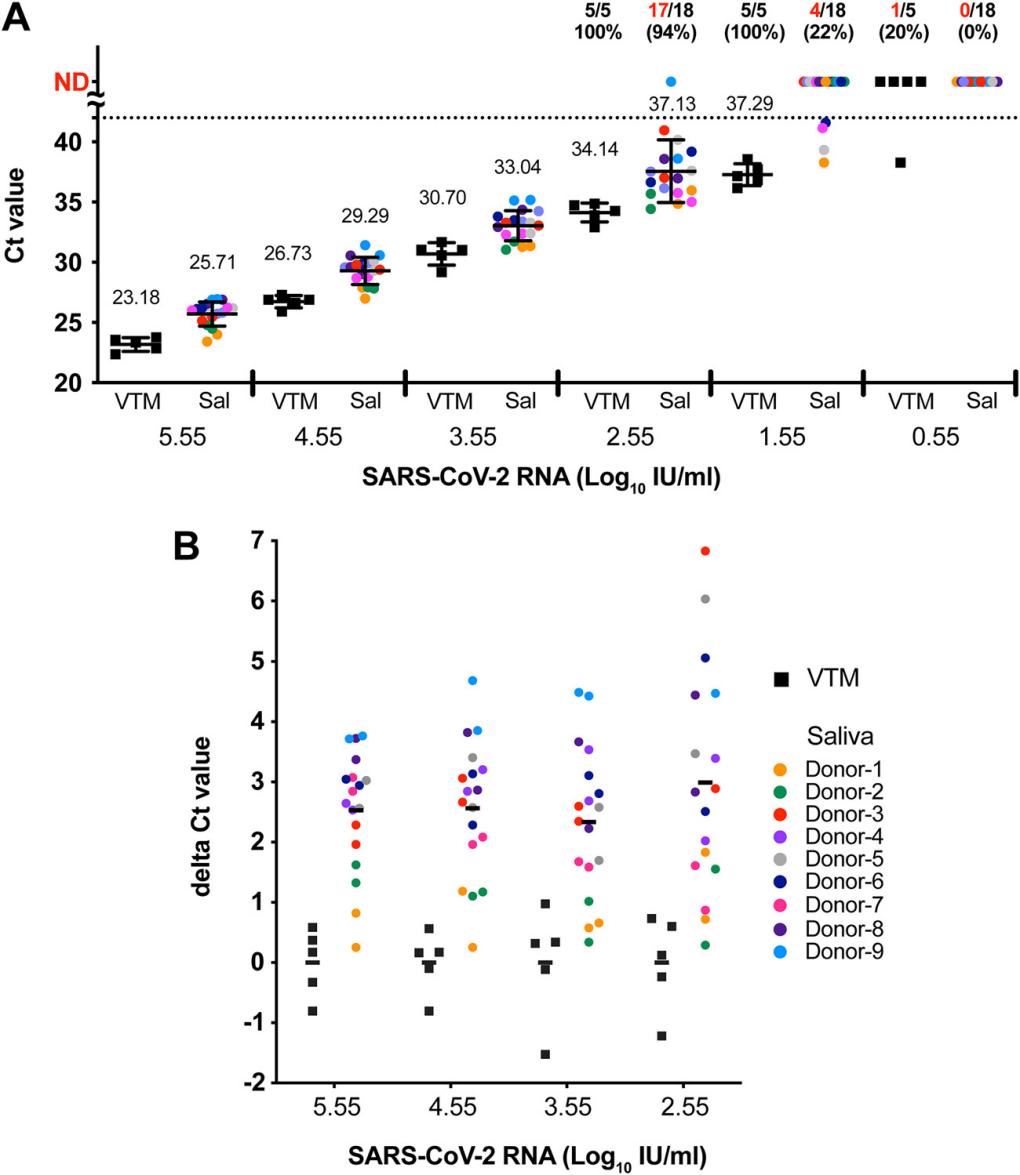
Effect of saliva on SARS-CoV-2 RNA detection. (A) Viral RNA levels of serially diluted inactivated SARS-CoV-2 virus samples (WHO international standard 20/146) supplemented with saliva (Sal) from different donors (*n* = 9) or viral transport medium (VTM). Each donor’s saliva was tested in duplicate. The dashed line indicates the detection limit of qPCR (*C_T_* value, 42). The viral RNA titers are shown as the mean ± SD. The numbers indicate the mean *C_T_* values and the proportion/percentage of positive samples for each test group. Negative samples were not included in the calculation of the means. Means were not calculated for groups in which more than half of the samples were negative. (B) Variation in the effect of individual saliva on SARS-CoV-2 RNA. The effect of saliva was evaluated by differences in *C_T_* values (delta *C_T_*) between each measured positive sample and the mean *C_T_* value of the VTM-treated group at the same RNA concentration. ND, under the detection limit.

To examine the effect of saliva on SARS-CoV-2 RNA detection, saliva samples obtained from nine healthy donors were individually mixed with different amounts (5.55 to 0.55 log_10_ IU/mL) of inactivated SARS-CoV-2 virus (WHO international standard 20/146). Subsequent viral RNA in the mixtures was quantified by qPCR. Viral transport medium (VTM) mixed with the inactivated virus served as the control. The saliva-treated samples showed an overall increase in the cycle threshold (*C_T_*) of 2 to 3 compared to the VTM control (Fig. 1A), and the extent of the increase in the *C_T_* values was consistent across the different input viral titers. To fit the data to the context of diagnosis, we evaluated the positive detection rate (*C_T_* value, <42) for different input viral titers. For mixtures with ≥2.55 log_10_ IU/mL inactivated virus, only 1 of the 72 saliva-treated samples tested negative (positive detection rate, 98.6%), whereas all VTM-treated samples tested positive. With lower input viral titers (1.55 and 0.55 log_10_ IU/mL), saliva could greatly affect viral RNA detection. For instance, with 1.55 log_10_ IU/mL (mean *C_T_* value, 37.29) SARS-CoV-2, only 4 of 18 saliva samples tested positive (22.2%), in contrast to 100% detection for the VTM-treated samples (Fig. 1A).

To further delineate the effect of individual saliva on viral RNA, the change in *C_T_* value (delta *C_T_*, the mean *C_T_* value of the VTM control subtracted from each saliva-treated sample) was calculated with different input viral titers (Fig. 1B). The extent of variation among the nine saliva donors was similar across the positive viral titers (3.55 to 5.55 log_10_ IU/mL), with delta *C_T_* values ranging from 0.25 to 4.68. Variation increased in saliva samples with lower virus titers (2.55 log_10_ IU/mL), with the maximum delta *C_T_* reaching 6.83 (Fig. 1B). With a low input viral load, differences between experimental duplicates of the same saliva sample also increased, which further implies the instability of saliva-treated viral RNA at low concentrations.

### Effect of storage temperature and time on saliva specimens.

**FIG 2:**
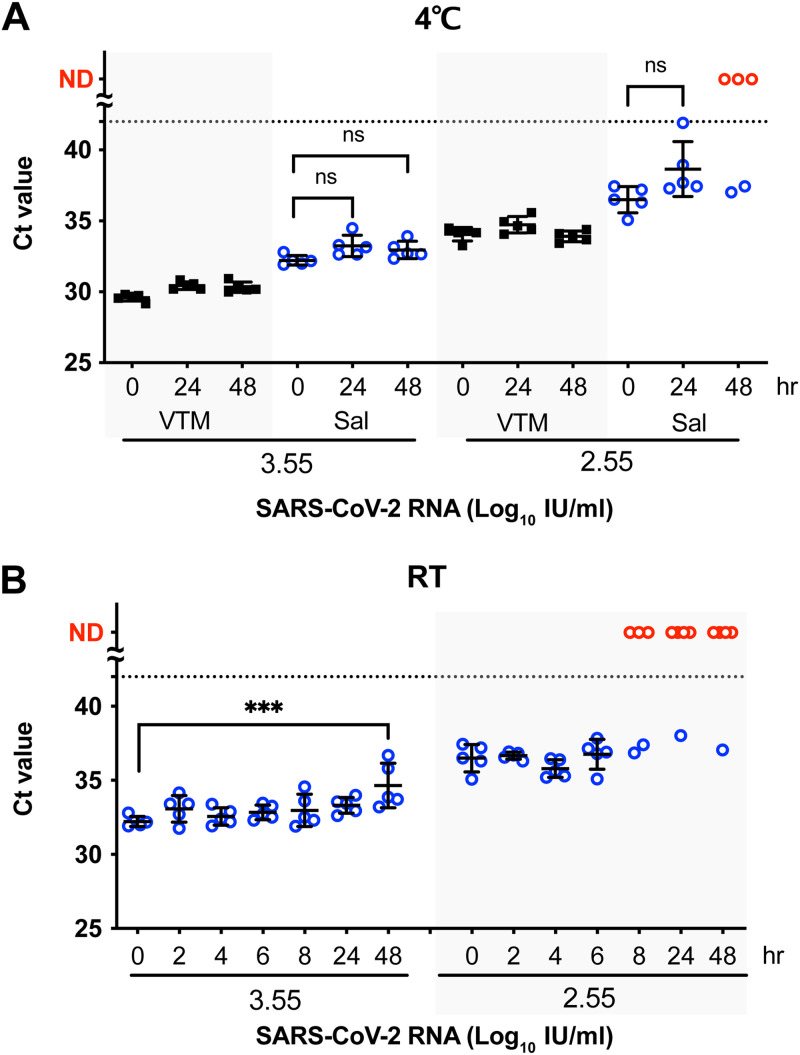
Effect of storage conditions on saliva-treated SARS-CoV-2. Inactivated SARS-CoV-2 virus samples were quantified after incubating with saliva or VTM for different lengths of time at 4°C (A) or room temperature (RT) (B). (A) Inactivated virus at high (3.55 log_10_ IU/mL) or low (2.55 log_10_ IU/mL) concentrations was quantified after incubating with saliva or VTM for 0, 24, and 48 h at 4°C. (B) High or low concentrations of inactivated virus were incubated with saliva for the indicated times at RT. RNA levels are shown as the mean ± SD. The dashed line indicates the detection limit of qPCR. Samples that tested negative are highlighted in red. Each group was tested with five replicates. ND, under the detection limit; ns, not significant. ***, *P* < 0.001; analyzed by one-way analysis of variance (ANOVA).

To test the effect of temperature on the viral RNA in saliva samples, inactivated SARS-CoV-2 was added to the pooled saliva from four different donors, and the samples were stored at 4°C or room temperature (RT) for the indicated times before RNA detection. With 3.55 log_10_ IU/mL of inactivated virus in VTM or saliva, incubation at 4°C for 24 or 48 h led to comparable levels of viral RNA (100% positive detection) (Fig. 2A). With a low virus titer (2.55 log_10_ IU/mL), only 40% of the saliva samples remained qPCR positive after 48 h at 4°C (Fig. 2A). We further examined the effect of saliva on viral RNA after incubation at RT. For saliva samples with a viral titer of 3.55 log_10_ IU/mL incubated at RT for 48 h, the *C_T_* value significantly increased, from 32.22 (0 h) to 34.66, but all the saliva samples tested positive (Fig. 2B). However, the virus was undetectable in more than half of the samples with a lower input viral titer (2.55 log_10_ IU/mL) after 8 h of incubation at RT (Fig. 2B). Thus, saliva samples could be stored at RT for up to 6 h without affecting the results of detection but should be stored at 4°C if a longer time is expected before testing.

### Naked RNA without a protein shell is more sensitive to saliva.

**FIG 3:**
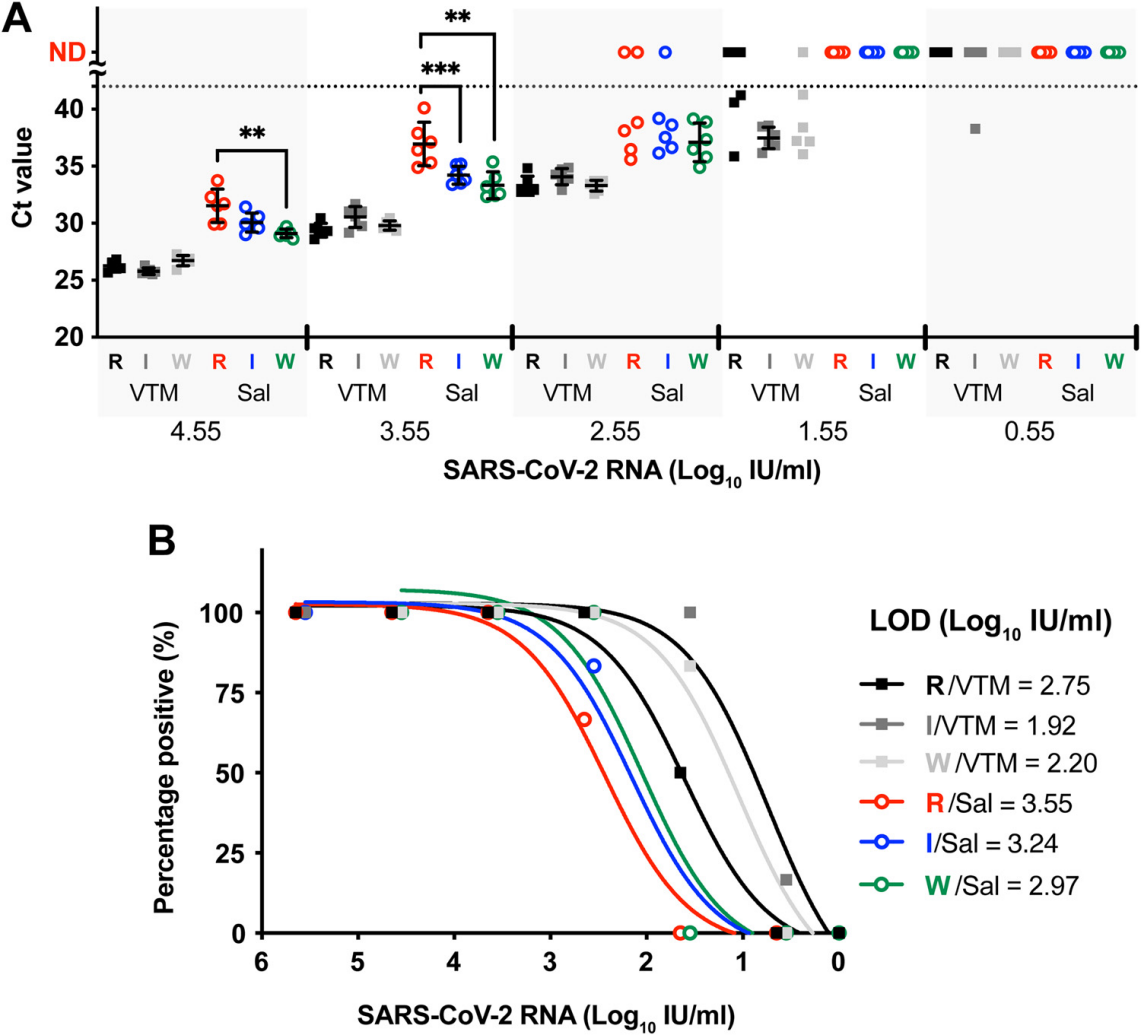
Stability of different SARS-CoV-2 samples spiked in saliva. (A) Serially diluted RNA extracted from SARS-CoV-2-infected mice (R), the international standard 20/146 (I), and the working standard 20/138 (W) was mixed with saliva (Sal, circle) or VTM (VTM, square). The viral RNA level in the mixture was quantified by qPCR. The international standard consisted of inactivated SARS-CoV-2, and the working standard consisted of SARS-CoV-2 RNA encapsulated lentiviral particles. The detected RNA titers (*C_T_* values) are shown as the mean ± SD. The dashed line indicates the detection limit of qPCR. ND, under the detection limit. **, *P* < 0.01; ***, *P* < 0.001; analyzed by one-way ANOVA. (B) Detection limits of saliva- or VTM-treated RNA samples. Limits of detection (LOD; 95% detection titer) were calculated with fitted lines of detection rate against SARS-CoV-2 viral RNA titers.

To understand the factors contributing to the reduced positive detection of saliva samples, we compared the qPCR results of three different samples: purified naked RNA, inactivated SARS-CoV-2 virus (international standard 20/146), and HIV-encapsulated SARS-CoV-2 RNA (working standard 20/138) after reacting with pooled saliva. The purified naked RNA was more sensitive to saliva treatment, with a significant increase of *C_T_* values (Fig. 3A) and a higher limit of detection (LOD) (Fig. 3B). However, the effect of saliva on the inactivated SARS-CoV-2 virus and HIV-encapsulated RNA was comparable; the pattern still held when the two standard samples were compared to the intact SARS-CoV-2 particles (see Fig. S1 in the supplemental material). These results suggest a protective role of the protein shell, regardless of the source, and an indirect role of salivary proteases in RNA degradation.

**FIG 4:**
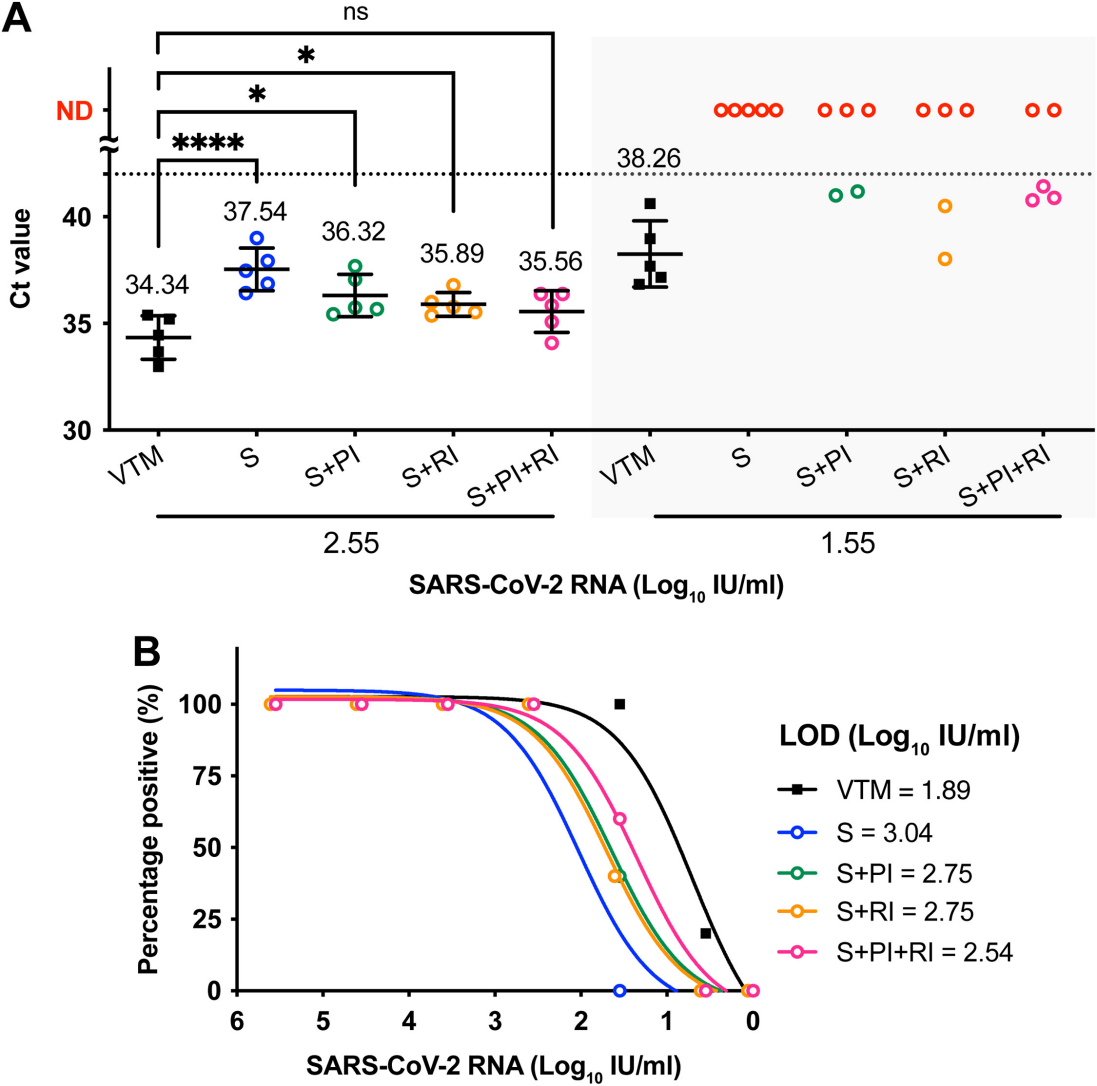
Supplementing an RNase inhibitor (RI) and a protease inhibitor (PI) improved the detection of saliva-treated SARS-CoV-2. (A) Different combinations of saliva (S), PI, and RI or viral transport medium (VTM) alone were added to the inactivated SARS-CoV-2 virus. The viral RNA levels of the mixture were quantified by qPCR. The dashed line indicates the detection limit of qPCR. ND, under the detection limit. Samples testing negative are highlighted in red. The numbers indicate the mean *C_T_* values of positive samples for each test group. ns, not significant; *, *P* < 0.05; ****, *P* < 0.0001; analyzed by one-way ANOVA. (B) Detection limits of RNA combined with saliva or inhibitors. Limits of detection (LOD; 95% detection titer) were calculated with fitted lines of detection rate against SARS-CoV-2 viral RNA titers.

To verify the roles of salivary RNases and proteases in degrading viral RNA, we evaluated the effect of protease inhibitor (PI) and RNase inhibitor (RI) treatment on saliva (S)-treated inactivated SARS-CoV-2. With the input virus at 2.55 log_10_ IU/mL, the addition of a PI or RI improved the saliva RNA detection, reducing the *C_T_* value from 37.54 (S) to 36.32 (S+PI) and 35.89 (S+RI) (Fig. 4A). Furthermore, with a combination of PI and RI treatment, the RNA detection in saliva did not significantly differ from that in the VTM control (*C_T_* value, 35.56 versus 34.34, respectively) (Fig. 4A). In addition, with an input viral load of 1.55 log_10_ IU/mL, applying a PI or RI increased the positive detection rate by 40%, and a PI and RI combined further increased the detection rate by 60%. Applying a PI or RI alone decreased the RNA detection threshold of saliva samples by ~2-fold, whereas applying a combined PI and RI decreased the threshold by ~3-fold (Fig. 4B). We found that PI or RI alone or a combination of both also significantly improved detection of the HIV-encapsulated SARS-CoV-2 RNA after saliva treatment (Fig. S2). These findings suggest the potential of PI/RI supplementation of saliva samples for better viral RNA detection.

## DISCUSSION

Saliva-based nucleic acid detection has been widely implemented in airports and schools for COVID-19 diagnosis because of its convenience, less invasiveness, and minimal demand on medical professionals (18–20). A study evaluating the infectivity of viruses in saliva samples showed that only a low proportion (11%) of samples contained infectious viruses (21), probably because of the enzymatic effects of components in the saliva. Saliva sampling may be a safe option for mass SARS-CoV-2 detection. However, the detection limit of the test and the direct effect of saliva on viral RNA had not been carefully examined. In this study, we used the well-defined WHO international standard 20/146 to characterize the effect of saliva on SARS-CoV-2 RNA detection. Saliva had a negative effect on SARS-CoV-2 RNA detection, with a mean increase of 2.64 in the *C_T_* value compared to the VTM control (Fig. 1A), similar to the *C_T_* value increase in a study of gamma-irradiated viruses (22). We further highlight the considerable variation in the effect of individual saliva, with increases in the *C_T_* value ranging from 0.25 to 6.83 compared to the VTM control (Fig. 1B). Numerous factors affect saliva secretion and composition, including age, sex, smoking, underlying diseases, and nutritional changes (23–30). How these factors affect the degradation of viral RNA by saliva requires further investigation.

SARS-CoV-2 RNA (>2.55 log_10_ IU/mL; *C_T_* value, <35) was detected in most of the saliva samples stored at 4°C for up to 24 h (Fig. 2A) or 6 h at RT (Fig. 2B), so the sample collection and laboratory workflow arrangement is feasible. Our findings agree with those of a previous study showing that low levels of viral RNA (*C_T_* value, >35) in patient saliva became undetectable after prolonged storage at RT or 4°C (21). Although low viral RNA in saliva could not be efficiently detected, the chance of an individual having such a low level of viral RNA and remaining infectious is low. Therefore, saliva-based SARS-CoV-2 detection can facilitate control measures, especially in the context of large-scale outbreaks.

A previous study demonstrated that at 4.4 log_10_ copies/mL, inactivated SARS-CoV-2 in saliva remained stable at 4°C for up to 7 days (22). However, our results show that >3.55 log_10_ IU/mL viral RNA in saliva remained stable for 2 days, whereas a lower concentration (2.55 log_10_ IU/mL) became undetectable after 2 days at 4°C storage (Fig. 2A). The seemingly longer detection duration in the previous study compared to our observations might be attributed to differences in the RNA quantification procedures, including the viral genes targeted by reverse transcription-quantitative PCR (RT-qPCR) and the RNA standard used to calculate the copy number. In addition, the effect of saliva may have been reduced in experiments with the same volume of saliva and inactivated viruses (1:1) mixed in the previous study, compared to the 9-fold volume of saliva used in our study (1:9 dilution).

SARS-CoV-2 lineage B.1.1.529, denoted the Omicron variant by WHO, has rapidly replaced the previously dominant Delta variant and has accounted for most global infections since the end of 2021 (31). SARS-CoV-2 can infect oral epithelial cells and small salivary glands (32). Recent studies of the Omicron variant further suggest that the new variant tends to invade the upper respiratory tract, in contrast to previous strains, which invade the lower respiratory tract or lung tissues (33, 34). A recent study of the Omicron variant further showed that compared to nasal midturbinate swabs, saliva provided higher sensitivity for Omicron virus detection (35). These studies highlight the applicability of SARS-CoV-2 saliva tests for future circulating strains.

Saliva has received increasing attention in recent years as a source of sampling for both transmissible and nontransmissible diseases, targeting nucleic acids (DNA or RNA), antigen, or antibody. Because of the complexity of human saliva, retrieving biomarkers such as RNA with good quantity and quality remains challenging (36). Our results, along with those from a previous study, demonstrate that the degradation of naked SARS-CoV-2 RNA can be delayed by adding an RNase inhibitor (37). We further demonstrate that both RNase and protease affect the viability and durability of SARS-CoV-2 in saliva. Overall, our findings provide practical guides for future saliva sampling for both COVID-19 and other lung-related diseases.

## MATERIALS AND METHODS

### SARS-CoV-2 RNA samples.

From the National Institute for Biological Standards and Control (NIBSC), we obtained the first WHO international standard (NIBSC code 20/146) and working standard (NIBSC code 20/138) for SARS-CoV-2 RNA. International standard 20/146 consists of acid-heat–inactivated SARS-CoV-2 (isolate England/02/2020; GenBank accession no. MW059036). After reconstitution, the standard contains 7.70 log_10_ IU/mL viral RNA plus approximately 1 × 10^5^ copies/mL human genomic DNA background. Working standard 20/138 consists of chimeric lentiviral particles with RNA from synthetic SARS-CoV-2 isolate Wuhan-1 packaged within HIV-1 particles. After reconstitution, the working standard contains 6.73 log_10_ IU/mL SARS-CoV-2 viral RNA. Also, naked RNA samples were extracted from SARS-CoV-2-infected mouse brains (38) and titrated with the international standard using international units. The intact SARS-CoV-2 (strain Wuhan-1) used in this study, originally clinical isolate TCDC#4 (hCoV-19/Taiwan/4/2020), was obtained from the Taiwan Centers of Disease Control (CDC). The virus was propagated in the biosafety level 3 (BSL3) facility of the Institute of Biomedical Sciences (Academia Sinica).

### Saliva samples and sample preparation.

Saliva samples were collected from nine healthy donors (donors 1 to 9) using wide-mouth DNase/RNase/protease-free containers. This study was approved by the institutional review board (IRB) of Academia Sinica (IRB no. AS-IRB-BM 22010). Donors were asked to abstain from eating and drinking for 2 h before sample collection. Viral transport medium (VTM; Copan Diagnostics), routinely used with nasopharyngeal swabs for virus diagnosis, was used as the control in this study. Saliva or VTM was mixed with specific titers (5.55, 4.55, 3.55, 2.55, 1.55, and 0.55 log_10_ IU/mL) of SARS-CoV-2 international standard before quantification by qPCR. The mixture was stored at two temperatures (room temperature and 4°C) and was quantified after specific durations of time.

### SARS-CoV-2 detection by real-time RT-qPCR.

The SARS-CoV-2 RdRp and N genes were detected by quantitative RT-PCR (RT-qPCR) using the Alinity m SARS-CoV-2 AMP kit (Abbott Laboratories, USA) according to the manufacturer’s instructions. Alinity m is an automated RT-qPCR detection system designed to automate the processes, from RNA extraction to reporting cycle threshold (*C_T_*) values. Before being loaded in the machine, 350 μL sample was mixed with an equal volume of lysis buffer. The *C_T_* value was determined by the system, and a positive result for the SARS-CoV-2 RNA detection was determined with a *C_T_* value of <42.

### RNase and protease inhibitor treatment.

Pooled saliva from four donors (donors 1, 4, 6, and 9) was supplemented with 2 U RNase inhibitor RNasin (40 U/μL; Promega), 1× cOmplete protease inhibitor cocktail (50×; Roche), or a combination of the two inhibitors in the presence of dithiothreitol (DTT). The saliva was then mixed with the SARS-CoV-2 international standard to reach 2.55 or 1.55 log_10_ IU/mL before RNA quantification.

### Detection limit of SARS-CoV-2 RNA.

A limit of detection (LOD) for a given treatment or condition to the viral RNA was defined as the corresponding RNA titer of 95% positive detection based on the fitted regression curve calculated using Prism 6 software. A regression curve was inferred by the serially diluted viral RNA titers and the resulting proportions of positive samples for each experimental group. The nonlinear regression equation used in this study is expressed as follows: *Y* = (top − bottom)/[1 + 10^(^*^X^*^−logIC50)^] + bottom. Here, *X* represents the RNA concentration in log_10_ IU/mL, *Y* represents the percentage of positive detection, and “top” and “bottom” denote the values at the plateaus.
