# Supplementary material for: Optimization and Improvement of qPCR Detection Sensitivity of SARS-CoV-2 in Saliva
Source: Microbiol Spectr. 2023 Apr 25;11(3):e04640-22. doi: 10.1128/spectrum.04640-22 (PMC10269702; doi:10.1128/spectrum.04640-22)
Supplement: Supplemental file 1 — Fig. S1 and S2. Download spectrum.04640-22-s0001.pdf, PDF file, 0.2 MB [file spectrum.04640-22-s0001.pdf]

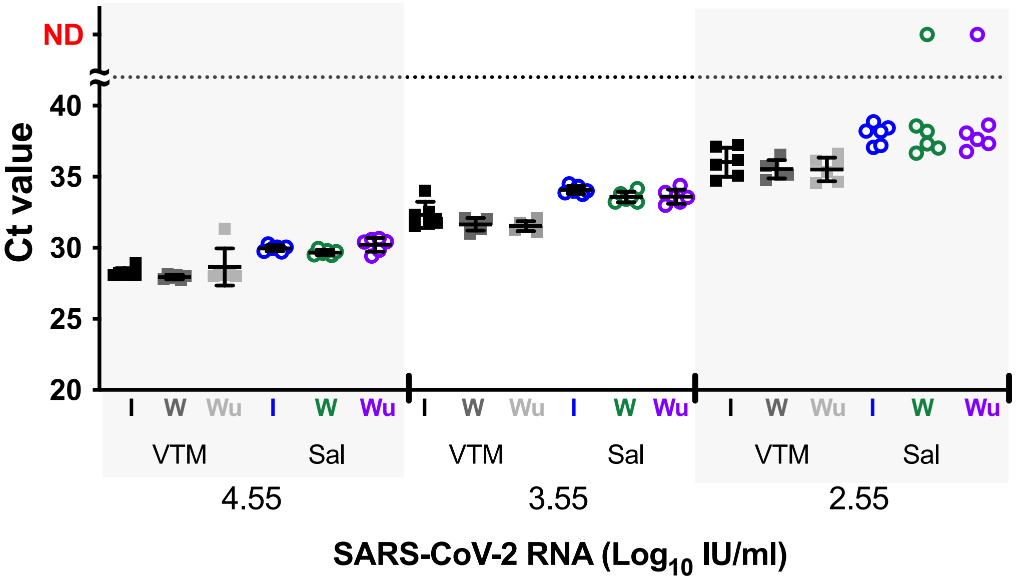

Figure S1. The stability of various SARS-CoV-2 samples spiked in either saliva or VTM was evaluated. The samples tested included serially diluted SARS-CoV-2 Wuhan strain (Wu), the international standard 20/146 (I), and the working standard 20/138 (W). The spiked samples were represented by circles when mixed with saliva (Sal) and squares when mixed with VTM. Quantification of viral RNA levels in the mixture was performed using qPCR. The international standard used in the study comprised acid-inactivated SARS-CoV-2, while the working standard was made up of SARS-CoV-2 RNA encapsulated lentiviral particles. The results of the RNA titers detected, represented by Ct values, are presented as mean ± SD, with the detection limit of qPCR shown as a dashed line. ND: under detection limit. Statistical analysis using one-way ANOVA showed no significant difference in the stability of the three types of SARS-CoV-2 samples spiked in saliva.

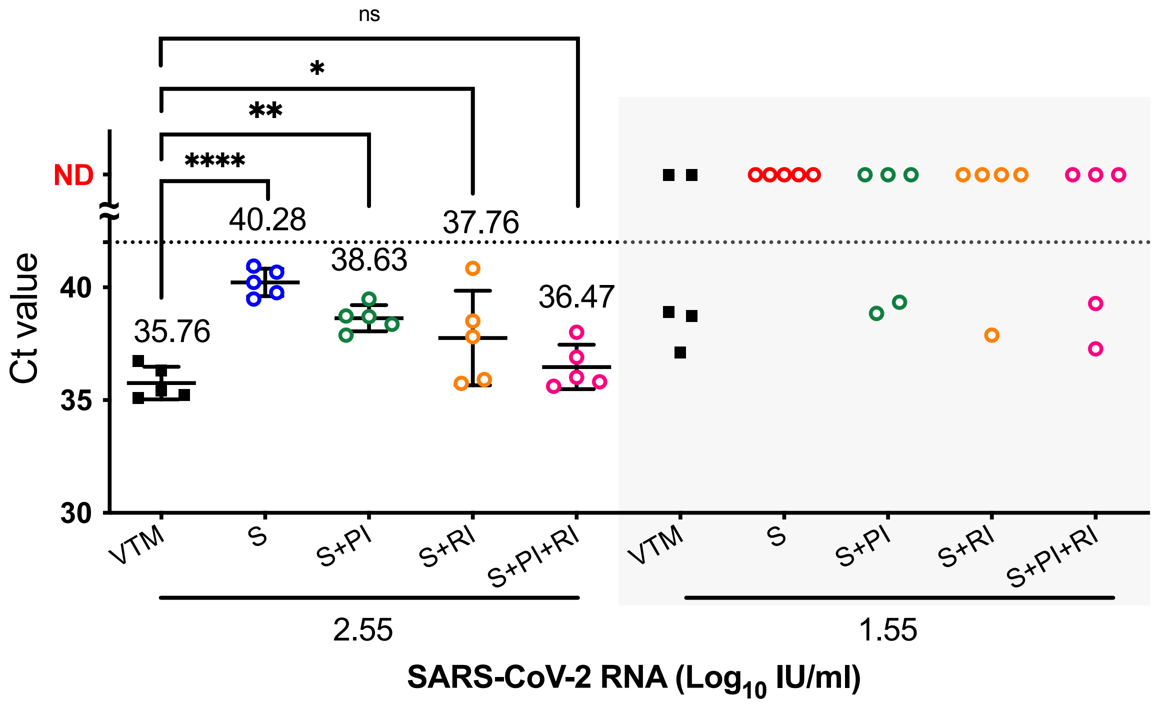

Figure S2. The detection of lentivirus-based SARS-CoV-2 particles treated with saliva (working standard, NIBSC code 20/138) was improved by the addition of a protease inhibitor (PI) and a RNase inhibitor (RI). Various combinations of saliva (S), PI, RI, or viral transport medium (VTM) alone were added to the inactivated SARS-CoV-2 virus. Quantification of viral RNA levels in the mixture was done by qPCR, with the detection limit of qPCR indicated by a dashed line. ND: under detection limit. Samples testing negative are highlighted in red. ns: not significant, *$p<0.05$, **$p<0.01$, ****$p<0.0001$, analyzed by one-way ANOVA.
